# Integrated Genomic Profiling of Newly Diagnosed and Relapsed Acute Myeloid Leukemia Identifies Driver Genes, Mutational Signatures, and Therapeutic Targets

**DOI:** 10.3390/cancers18101532

**Published:** 2026-05-09

**Authors:** Harsh Goel, Avanish Kumar Pandey, Anshul Arya, Rahul Kumar, Rakesh Kumar, Harshita Makkar, Ravi Kumar Majhi, Sujata Bhattacharya, Jay Singh, Mohit Kumar Divakar, Payal Vasudeva, Saran Kumar, Anita Chopra, Amar Ranjan, Jagdish Prasad Meena, Aditya Kumar Gupta, Ganesh Kumar Viswanathan, Atul Batra, Goura Kishor Rath, Showket Hussain, Garima Jain, Aroonima Misra, Ekta Rahul, Sameer Bakhshi, Pranay Tanwar

**Affiliations:** 1 Laboratory Oncology Unit, Dr. B.R.A. Institute Rotary Cancer Hospital, All India Institute of Medical Sciences, New Delhi 110029, India; harshgoel@aiims.edu (H.G.);; 2 Division of Pediatric Oncology, Department of Pediatrics, All India Institute of Medical Sciences, New Delhi 110029, India; 3 Department of Medical Oncology, Dr. B.R.A. Institute Rotary Cancer Hospital, All India Institute of Medical Sciences, New Delhi 110029, India; 4 Kusuma School of Biological Sciences, Indian Institute of Technology Delhi, New Delhi 110016, India; 5 Department of Hematology, All India Institute of Medical Sciences, New Delhi 110029, India; 6 Department of Radiotherapy, Dr. B.R.A. Institute Rotary Cancer Hospital, All India Institute of Medical Sciences, New Delhi 110029, India; 7 Division of Molecular Oncology, National Institute of Cancer Prevention & Research, Noida 201301, India; 8 Division of Digital Health, National Institute for Research in Digital Health and Data Science, New Delhi 110029, India; 9 Division of Oncology, National Institute of Child health and Development Research, New Delhi 110029, India; 10 Department of Pathology, All India Institute of Medical Sciences, New Delhi 110029, India

**Keywords:** acute myeloid leukemia, whole-exome sequencing, mutational signatures

## Abstract

Background/Objectives: Acute myeloid leukemia (AML) is a hematologic malignancy of substantial genetic heterogeneity that exhibits clonal growth and blocked differentiation of myeloid progenitor cells in the bone marrow (BM). Genetic alterations play a vital role in the progression, initiation, and recurrence of AML. The aim of this study was to identify the somatic mutational landscape, pathway perturbations, mutational signatures, and druggability of baseline (at diagnosis) and relapsed AML to determine possible treatment options. Methods: Between 2020 and 2026, 120 diagnostic BM or PB samples were prospectively collected from baseline (at diagnosis) AML patients at AIIMS, New Delhi. WES was conducted of 10 BM samples from five baseline (at diagnosis) and five relapsed patients with AML. Somatic variations were identified by GATK-Mutect2 and annotated by ANNOVAR. Driver genes and pathways were analyzed using Maftools, OncodriveCLUST, and clusterProfiler. Extraction of mutational signatures was performed with the help of SigProfilerExtractor, and evaluation of drug–gene interactions was carried out with the help of DGIdb. In addition, RT-PCR was performed to estimate the expression level of *TET1*. The recurrent *TET1* variation was validated using Sanger sequencing and PCR amplification. Results: Missense mutations were the most common variant type in the cohort, and C > T transitions were the predominant nucleotide substitution pattern. There were recurrent mutations in core AML driver genes, including *TET1*, *FLT3,* and *TP53*, and relapsed samples demonstrated increased involvement and complexity of the signaling system. Pathway analysis revealed widespread dysregulation of carcinogenic networks, including *RTK-RAS*, *WNT*, *TP53*, and *PI3K* signaling. Mutational signature analysis identified COSMIC SBS5, SBS8, and SBS40, which are associated with mechanisms involving oxidative damage. A large number of actionable targets were identified through druggability screening, particularly involving epigenetic regulators and kinase-associated pathways. RT-PCR analysis also supported altered *TET1* expression in AML samples. The *TET1* A256V variant was detected and experimentally validated. Conclusions: This study highlights the somatic mutational landscape of baseline (at diagnosis) and relapsed AML and identifies recurrent driver genes, altered signaling pathways, mutational signatures, and actionable targets with possible therapeutic relevance. The integration of mutational and expression analyses further supports a potential role for *TET1* in AML biology, although the functional significance of specific variants such as A256V remains uncertain.

## Simple Summary

AML is a serious blood cancer that is caused by a proliferation of immature white blood cells in the bone marrow (BM). The condition is brought about by genetic alterations that disrupt normal cell division, cell growth, and DNA repair. In this study, we investigated mutated genes in BM samples from patients with newly diagnosed or relapsed AML. We determined some of the important genes and biological pathways that are linked to AML development and resistance to treatment. Moreover, we identified genes that can be targeted by current or experimental treatment. Finally, we experimentally validated the recurrent *TET1* variant identified in the cohort. These findings can help clinicians learn more about the development of leukemia, and they may help develop more effective therapies.

## 1. Introduction

Acute myeloid leukemia (AML) involves unregulated proliferation and differentiation of myeloid progenitor cells, resulting in impaired hematopoiesis and bone marrow failure [1,2]. AML demonstrates a higher incidence in males compared to females [3]. The most prevalent type of acute leukemia in adults is AML, and it is the eleventh most frequent cause of cancer-related mortality in the world, with a projected 15% increase in incidence in the next three decades [4,5]. Despite established treatment modalities such as induction chemotherapy, consolidation therapy, and allogeneic stem cell transplantation, long-term survival remains suboptimal, particularly in older patients and those with relapsed disease [6,7,8]. Despite the fact that most patients are able to reach full remission after induction treatment, a significant number later relapse, which emphasizes the necessity to develop better treatment methods [9,10,11]. The mechanisms of pathogenesis of AML are predetermined by a variety of genetic mutations that influence signal transduction, epigenetic regulation, DNA repair, and apoptosis. Due to its high degree of biological heterogeneity, comprehensive genomic characterization is essential for proper risk stratification, monitoring of the disease, minimal residual disease assessment, and determining the mechanism of therapeutic resistance [12,13]. Emerging evidence suggests that resistance in acute leukemia may involve not only the acquisition of new driver mutations, but also alterations in protein stability and degradation pathways, as well as secondary mutations affecting non-rearranged alleles. These mechanisms highlight the biological complexity of relapse and underscore the importance of comprehensive genomic profiling to identify relapse-specific drivers [14]. With the invention of next-generation sequencing (NGS), our knowledge of AML biology has greatly improved through findings of repeated somatic mutations in several functional gene categories, including epigenetic regulators, spliceosome components, metabolic enzymes, and signaling molecules. Recurrent mutations in *FLT3*, *NPM1*, *KIT*, *CEBPA*, *DNMT3A*, *IDH1*/2, *TET2*, *ASXL1*, *EZH2*, *MLL,* and *TP53* have been identified in large-scale genomic studies and play a central role in the pathogenesis and development of leukemia [15,16]. These changes include interference with epigenetic regulation as a characteristic feature of AML. Mutations in genes encoding DNA methylation regulators (e.g., *DNMT3A*, *TET2*, *IDH1/2*), chromatin modifiers (e.g., *ASXL1*, *EZH2*), and transcription factors (e.g., *CEBPA*, *RUNX1*) are frequently observed in AML and contribute to alterations in chromatin structure and transcriptional programs, ultimately influencing cell fate decisions and leukemic transformation [17,18]. These findings have transformed the concept of AML as a cytogenetically determined condition into an entity with an elaborate and dynamic structure of mutations [19,20]. Notably, mutational profiles are currently offering prognostic data and have enhanced risk stratification, in addition to indicating actionable molecular targets to facilitate precision medicine strategies in the treatment of AML [21,22,23]. Clonal evolution and enhanced genetic complexity are known to be related to relapsed AML [24]. Longitudinal sequencing has demonstrated both enrichment of high-risk mutations, such as those in *TP53*, and expansion of treatment-refractory subclones between diagnosis and relapse [25]. Subclonal driver mutations arise and are acquired in the development of diseases, leading to therapeutic failure and relapses [26,27]. In addition, mutational signature analyses have shown that endogenous mutagenic activities, including spontaneous cytosine deamination and defects in DNA repair pathways, are leading contributors to the mutational burden in AML [28,29]. Since AML is geographically and ethnically diverse, and there is a lack of genomic data in Indian patient groups, there is an urgent necessity to develop population-specific molecular profiling. In the present work, whole-exome sequencing of ten Indian AML cases, five with baseline disease (at diagnosis) and five with relapsed disease, was performed on bone marrow aspirates to comprehensively study the mutational landscape of the coding regions, explore recurrent mutations in key driver genes, identify mutational signatures, and define potential drug–gene interactions networks.

## 2. Materials and Methods

### 2.1. Patient Sample Collection and Exome Sequencing

Bone marrow aspirates were obtained from 120 AML patients, after obtaining written informed consent. The study was performed following the Declaration of Helsinki and, with the approval of the Institutional Ethics Committee of the All India Institute of Medical Sciences (AIIMS), New Delhi, India (Approval No. IECPG-71/27.01.2021). Whole-exome sequencing (WES) was performed on a subset of paired samples (*n* = 5), comprising matched baseline (at diagnosis) and relapsed samples from the same patients (*n* = 5 pairs), to enable intra-patient comparative analysis of clonal evolution. Clinical data including age, cytogenetic profiles, and treatment history were collected where available; however, these variables were not included in the present genomic analysis. Ficoll-Paque density gradient centrifugation was used to isolate mononuclear cells. A DNeasy Blood and Tissue kit (Qiagen, Hilden, Germany) was used to extract the genomic DNA according to the instructions of the manufacturer. Quantification of DNA concentration was performed using Qubit fluorometrics (Thermo Fisher, Waltam, MA, USA), and DNA integrity and purity were measured on an Agilent TapeStation 4200 system (Agilent Technologies, Santa Clara, CA, USA). Samples with A260/280 ratios of 1.8 to 2.0 and intact DNA profiles were selected for library preparation. An Agilent SureSelect Human All Exon V7 kit (Agilent Technologies, Santa Clara, CA, USA) was used to prepare whole-exome libraries. An Illumina NovaSeq 6000 (Illumina, San Diego, CA, USA) was used to sequence the samples (paired-end sequencing, 2 × 150 bp), with an average sequencing depth of 120× and a coverage of target regions of over 95% at 30× depth per sample.

### 2.2. Somatic Variant Calling and Annotation

FastQC (v0.12.1) was used to quality check raw sequencing reads, and Trimmomatic (v0.39) was used to trim adapter sequences. BWA-MEM (v0.7.17) was used to align high-quality reads to the human reference genome (GRCh38/hg38). Post-alignment processing, including duplicate marking, sorting, and base quality score recalibration, was performed using the Genome Analysis Toolkit (GATK, v4.4), following best-practice guidelines. Somatic single-nucleotide variants (SNVs) and small insertions/deletions were identified using Mutect2 (GATK) and filtered using FilterMutectCalls. Variant annotation was performed using ANNOVAR (version 2020-06-08) based on the RefSeq, COSMIC (v96), and ClinVar databases. The maftools package (v. 4.3.1) in R (v. 4.3.1) was used to generate and analyze MAF files. SNV classes, variant classifications, and mutation types were summarized, and mutation burden per sample was visualized using bar plots and boxplots. Oncoplots were generated to represent the overall mutational profile of AML samples. In the absence of matched normal samples, stringent bioinformatic filtering strategies were applied to distinguish somatic variants from germline variants, including removal of variants present in population databases (e.g., gnomAD, 1000 Genomes, and ExAC) at appreciable allele frequencies and filtering based on allele frequency and functional relevance. Variants were further filtered using a minimum variant allele frequency (VAF) threshold of ≥5% and adequate read depth to ensure high-confidence variant calls. Despite these filtering strategies, the absence of matched germline controls may result in residual germline variants being retained. Variants with high population allele frequencies were interpreted with caution. Variant interpretation was performed in accordance with ACMG/AMP guidelines, incorporating population frequency, computational predictions, and available clinical annotations. Variant allele frequencies (VAFs) were calculated for all variants and used to support variant prioritization and interpretation.

### 2.3. Identification of AML Driver Genes

OncodriveCLUST was used to identify putative AML driver genes according to mutation clustering and position. Candidate drivers were those genes which showed statistically significant hotspot clustering (adjusted q = false discovery rate q). Permutation-based clustering tests were used to evaluate statistical significance and applied in OncodriveCLUST. Binary mutation matrices (presence/absence of mutations per gene per sample) were built, and hierarchical clustering was performed with the ComplexHeatmap R package to compare driver mutation profiles between baseline (at diagnosis) and relapsed AML cases.

### 2.4. Pathway and Functional Enrichment Analysis

The recurrently mutated genes were mapped to oncogenic and signaling pathways in the MSigDB (v7.5.1), Reactome, and KEGG databases. Bar plots and treemaps were used to visualize pathway-level enrichment of mutations. The clusterProfiler R package was used to conduct a Gene Ontology (GO) enrichment analysis, including biological process (BP), molecular function (MF), and cellular component (CC) categories, as well as KEGG pathway enrichment analysis. Benjamini–Hochberg correction was applied, and pathways with adjusted *p*-values (FDR) < 0.05 were considered statistically significant.

### 2.5. Mutational Signature Analysis

MAF SNVs were placed in mutational patterns, which were analyzed with SigProfilerExtractor (v.1.2.6) and MutationalPatterns (v.3.18) to extract somatic SNVs. Non-negative matrix factorization was used to extract the signature. Comparisons between extracted signatures and COSMIC mutational signatures (v3.3) were performed by evaluating cosine similarity scores. The proportion of the contribution of each mutational signature to the samples was represented in a stacked bar plot, and similarities between inter-sample signatures are presented in the form of a cosine similarity heatmap. The annotated Cosmic SBS signatures which were most similar were annotated together with their reported etiologies. Only mutational signatures contributing ≥ 5% of the total mutational burden were considered for downstream analysis.

### 2.6. Drug–Gene Interaction and Therapeutic Target Analysis

The Drug Gene Interaction Database (DGIdb v4.3.0) was used to identify potentially druggable genes. Known and predicted drug interactions were screened using AML-associated genes that had somatic mutations. The interaction networks of drugs and genes were plotted with the help of igraph (v. 2.0.3) and Cytoscape (v. 3.10.2). Bar plots were used to summarize the number of known inhibitors per gene and the functional classifications. The UniProt annotations and DGIdb functional categories were used to classify genes as kinases, methyltransferases, transcription factors, or tumor suppressors. Only curated and pharmacologically relevant drug–gene interactions were considered for downstream analysis.

### 2.7. Variant Visualization, PCR Amplification, and Sanger Sequencing Validation

The mutation distribution and protein domain architecture of *TET1* were plotted using the lollipopPlot function of maftools. A QIAamp DNA Mini kit (Qiagen, Hilden, Germany) was used to isolate genomic DNA from chosen samples of bone marrow from patients with AML. PCR was performed to amplify the *TET1* exon 1 region that contains the A256V variant, with the following primers: Forward: 5′-CCCACTGGTCGTAGCCAAAT-3′; Reverse: 5′-CTGGAGGTGGGGTAGCAATC-3′. PCR was performed with Taq DNA Polymerase (Thermo Fisher Scientific, Waltman, MA, USA ) in a 25 µL volume under the following standard conditions of cycling: first denaturation at 95 °C for 30 s, 35 cycles of denaturation at 95 °C for 30 s, annealing at 58 °C for 30 s, and extension at 72 °C for 45 s. The PCR products were separated in a 1.5% agarose gel and stained with ethidium bromide. Amplicons were then purified and subjected to Sanger sequencing using an ABI 3730 DNA Analyzer (Applied Biosystems, Foster City, CA, USA). Chromatograms were sequenced, and Chromas software (v. 2.6.6) was used to align the sequenced fragments to the *TET1* reference transcript (NM_030625). Both the Integrative Genomics Viewer (IGV) exome sequenced read alignment and Sanger sequencing chromatograms confirmed the *TET1* c.767C: T (p.A256V) mutation.

### 2.8. RNA Isolation and Quantitative Real-Time PCR

Total RNA was extracted from bone marrow mononuclear cells using TRIzol reagent (Thermo Fisher Scientific, USA) according to the manufacturer’s instructions. RNA concentration and purity were determined using a NanoDrop spectrophotometer (Thermo Fisher Scientific, Waltham, MA, USA), and integrity was assessed by agarose gel electrophoresis. Complementary DNA (cDNA) was synthesized from 1 µg of total RNA using a High-Capacity cDNA Reverse Transcription kit (Applied Biosystems, Foster City, CA, USA). Quantitative real-time PCR (qRT-PCR) was performed using SYBR Green Master Mix (Applied Biosystems, Foster City, CA, USA) on a QuantStudio Real-Time PCR System (Applied Biosystems, Foster City, CA, USA) to evaluate the expression levels of *TET1*, with GAPDH used as an endogenous control. The thermal cycling conditions consisted of an initial denaturation step at 95 °C for 10 min, followed by 40 cycles of denaturation at 95 °C for 15 s and annealing/extension at 60 °C for 1 min. Relative gene expression was calculated using the 2^−ΔΔCt^ method. All reactions were performed in triplicate, and no-template controls were included to ensure specificity.

### 2.9. Statistical Analysis

All statistical tests and data visualization were carried out using R software (v4.3.1). Unless indicated to the contrary, *p*-values were corrected using the Benjamini–Hochberg correction method, and adjusted *p*-values below 0.05 were considered to be statistically significant.

## 3. Results

### 3.1. Somatic Mutation Landscape of the AML Cohort

**Figure 1:**
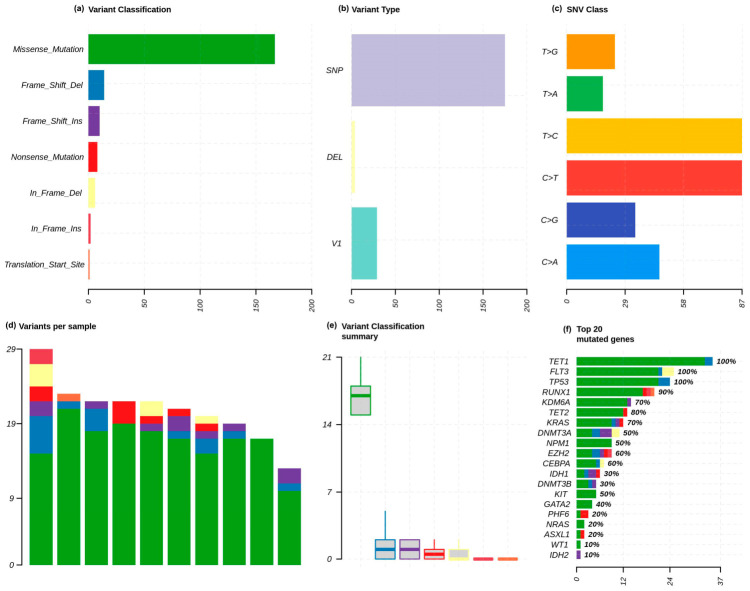
Overview of the characteristics of somatic variants in the AML cohort. (**a**) Classification of variants with missense mutations (dominant variant type), deletions/insertions leading to frameshifts, deletions/insertions causing nonsense mutations, and in-frame indels. (**b**) Variant type distribution showing SNPs as the predominant variant. (**c**) SNV substitution maps showing that C > T and T > C transitions are predominant. (**d**) Variants per sample showing moderate heterogeneity (10–29 variants). (**e**) Variant classification summary showing consistent missense predominance. (**f**) Top frequently mutated genes: *TET1*, *TP53*, *RUNX1*, *KDM6A*, *TET2*.

Analysis of somatic variants showed that missense mutations were the most common type of coding changes throughout the AML cohort, followed by frameshift insertions/deletions, nonsense mutations, and in-frame indels (Figure 1a). In line with this, the majority of the variants detected were single-nucleotide variants (SNVs), with a lesser percentage of variants comprising insertions and deletions (Figure 1b). Analysis of SNV substitution patterns showed that there was a great majority of C > T transitions, then T > C transitions, followed by C > A transversions (Figure 1c). The inter-patient heterogeneity was moderate, with an average of 10 to 29 somatic variants per sample (Figure 1d). In spite of this heterogeneity, missense mutations were always responsible for the majority of the alterations in most cases, as confirmed by variant summary classification (Figure 1e). Recurrence analysis at the gene level revealed that there were some commonly mutated genes in the group (Figure 1f). Recurrent alterations were observed in genes such as *TET1*, *FLT3*, and *TP53*; however, given the limited cohort size, these frequencies should not be interpreted as being representative of broader AML populations. There were also recurrent mutations in *KRAS*, *DNMT3A*, *NPM1*, *EZH2*, *CEBPA,* and *IDH1* with other, less frequently altered, genes including *ASXL1*, *PHF6*, *WT1*, *NRAS,* and *IDH2*. Overall, epigenetic regulator, signaling molecule, and canonical AML driver gene alterations were enriched in the somatic mutation landscape.

### 3.2. Recurrent Mutations and Distinct Genomic Patterns in Baseline and Relapsed AML

**Figure 2:**
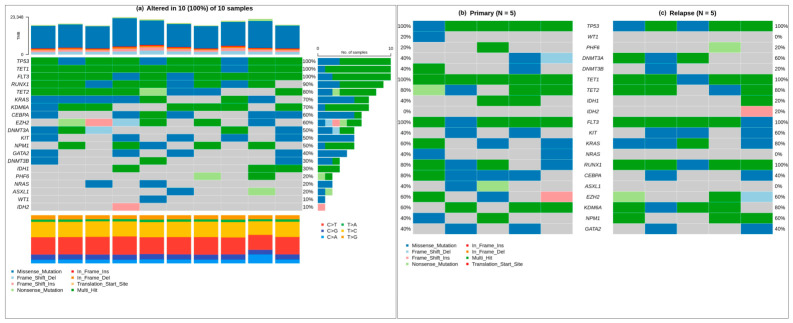
Patterns of recurrent mutations in AML, with baseline–relapsed comparison. (**a**) Cohort-wide mutation map reporting gene-scale frequencies, as well as the classes of mutation. (**b**) Mutational profile of baseline (at diagnosis) AML cases (*n* = 5), highlighting recurrent mutations in transcriptional and epigenetic regulators. (**c**) Relapsed AML sample mutation profiles (*n* = 5) revealed mutation enrichment in signaling and chromatin-modifying disease recurrence–linked genes.

Analysis of cohorts showed widespread somatic mutation recurrence in the major leukemogenic pathways (Figure 2a). High-frequency alterations in *TP53*, *TET1*, and *FLT3* were observed in this cohort; however, these frequencies likely reflect sampling variability and should not be interpreted as being representative of larger AML populations. Other recurrent mutations were observed in *CEBPA*, *EZH2*, *DNMT3A*, *KIT*, and *NPM1,* whereas lower-frequency events were seen in *GATA2*, *PHF6*, *NRAS*, *ASXL1*, *WT1*, *IDH1*, and *IDH2*. In all samples, the missense mutations were dominant, and C > T and T > C exchanges were enriched in mutations associated with AML. Disease-based stratification indicates that there is a difference between baseline (at diagnosis) and relapsed AML. Baseline (at diagnosis) AML cases (Figure 2b) were characterized by frequent mutations in *RUNX1*, *TET2*, *TET1*, *EZH2*, and *CEBPA*, indicating early impairment of transcriptional regulation and epigenetic control. In contrast, relapsed AML samples (Figure 2c) exhibited a more complicated mutational landscape, characterized by increased frequencies of *KRAS*, *KIT*, *DNMT3A*, *KDM6A*, and *IDH2* mutations. An apparent enrichment in *KRAS* mutations was observed in relapsed samples; however, this observation should be interpreted cautiously, due to the limited sample size.

These changes likely reflect clonal evolution driven by therapeutic pressure and expansion of resistant subclones, leading to proliferation of resistant subclones containing mutations in signaling and chromatin-modifying genes. Overall, such results suggest that there is a common AML driver mutation core with divergent evolutionary patterns between diagnostic and relapsed disease.

### 3.3. Distribution and Functional Clustering of AML Driver Gene Mutations

**Figure 3:**
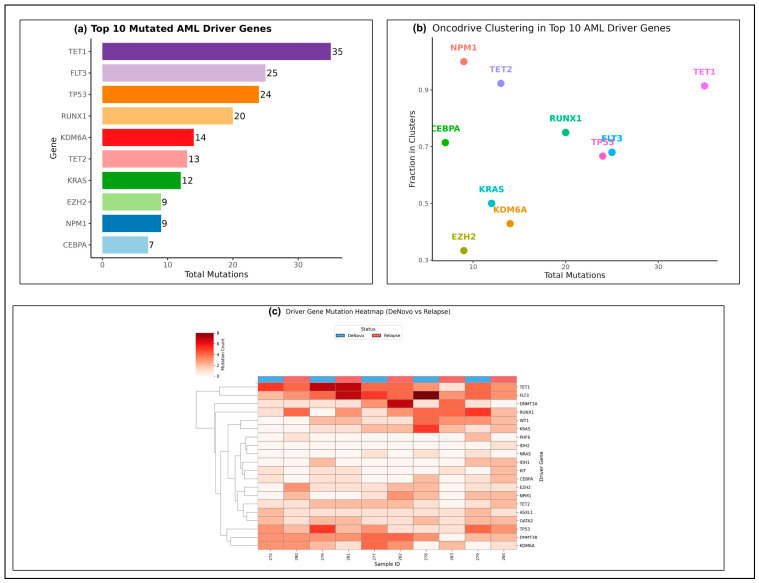
Driver gene mutation profiles and clustering in AML. (**a**) Number of mutations in the top 10 AML driver genes, with the highest recurrence rates seen in *TET1*, *FLT3*, *TP53*, and *RUNX1*. (**b**) Oncodrive clustering of driver genes, identifying functionally enriched mutational hotspots. (**c**) Heatmap of the distribution of driver gene mutations across samples, which shows the heterogeneity and clustering of mutational patterns among cases.

To investigate the role of critical leukemogenic drivers, we quantified the total number of somatic mutations in known AML driver genes across all samples (Figure 3a). *TET1* exhibited the highest number of mutations (*n* = 35), followed by *FLT3* (*n* = 25), *TP53* (*n* = 24), and *RUNX1* (*n* = 20). These values represent the total mutation counts aggregated across the 10 analyzed samples rather than the number of affected patients. Other frequently mutated driver genes were *KDM6A* (*n* =14), *TET2* (*n* = 13), and *KRAS* (*n* = 12). Recurrent deletion mutations were also observed at low frequencies in *EZH2*, *NPM1*, and *CEBPA*, all of which are still central to AML pathogenesis. The Oncodrive clustering analysis indicated that there was positional clustering in several high-impact genes (Figure 3b). *TET1*, *NPM1*, *TET2,* and *RUNX1* had very strong clustering, which suggested preferential mutation at functional locations. Strong clustering was also observed in *TP53* and *FLT3*, and these mutations are expected to be pathogenic. Mutations in *KRAS*, *KDM6A,* and *EZH2*, on the other hand, were moderately clustered, indicating a mixture of hotspot and scattered mutation patterns. The analysis of driver gene mutations in samples (Figure 3c) indicated that there was heterogeneous engagement of driver genes in each of the patients. The samples were characterized by different degrees of accumulation of mutations in each of the major driver genes, i.e., *TET1*, *FLT3*, *DNMT3A*, *RUNX1*, and *KDM6A*, with relapsed samples tending to have more mutations in several of the major driver genes. Hierarchical clustering showed that samples were clustering together based on common patterns of driver mutations, with different clusters containing samples with higher epigenetic regulator mutation signals as compared to samples with higher signaling gene mutation signals. This suggests the existence of some biological subgroups among the cohort. Combined, these analyses illustrate that AML in this cohort is typified by massively recurrent mutations in the core driver genes, robust functional clustering of these mutations, and heterogeneous sample-wide distribution, which represents varied leukemogenic pathways.

### 3.4. Pathway-Level Dysregulation Revealed by Somatic Mutation Analysis

**Figure 4:**
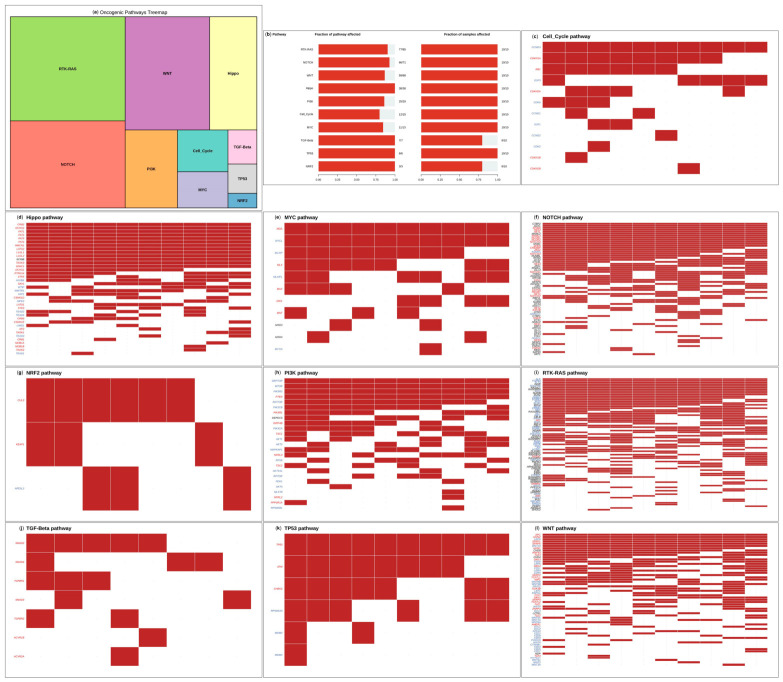
Somatic alterations detected at the pathway level in the AML cohort. (**a**) Treemap showing the relative contributions of the key mutated oncogenic pathways affected by mutations, including the *RTK–RAS*, *WNT*, *NOTCH*, *PI3K*, *Hippo*, *TGF-β*, *cell cycle*, *TP53*, *MYC*, and *NRF2* pathways. (**b**) Bar graph of the distribution of samples with mutations in each pathway, showing the degree of pathway involvement in the cohort. (**c**–**l**) Pathway-specific mutation maps of the *Hippo*, *MYC*, *NOTCH*, *RTK-RAS*, *NRF2*, *PI3K*, TGF-b, *TP53*, *cell cycle*, and *WNT* pathways, which indicate the distribution of mutated genes and the heterogeneity of pathway disruption among samples.

Pathway analysis revealed that somatic mutations converge in several pathways related to oncogenesis and regulation. A treemap representation of pathway involvement revealed broad disruption of major cancer-associated pathways, with the *RTK–RAS*, *WNT*, *NOTCH*, *PI3K*, *Hippo*, *TGF-β*, *cell cycle*, *TP53*, *MYC*, and *NRF2* pathways prominently affected (Figure 4a). Of these, disruption of the *RTK-RAS* pathway was most common, as indicated by frequent mutation of *FLT3*, *KRAS*, and *KIT*. The proportion of genes and samples affected in each pathway was quantitatively analyzed, and the results showed that the *RTK-RAS* and *NOTCH* pathways exhibited the highest levels of disruption, followed by the *WNT* and *Hippo* pathways (Figure 4b). There was also significant disruption of the *cell cycle* regulation, chromatin dynamics, and transcriptional control pathways, as manifested by changes in the *TP53* and *MYC* pathways. These data demonstrate that AML in this cohort is characterized by a sophisticated and multi-layered structure of pathway deregulation, with parallel deregulation of a number of interrelated biological axes. Detailed pathway-specific heatmaps were also available, which gave more information regarding the localization of mutations in different pathways (Figure 4c–l). The changes in the *Hippo* pathway were widespread in upstream elements, which might have consequences regarding proliferation control and cell fate choice. The *MYC* pathway exhibited numerous repetitive changes that acted on regulators of *MYC* stability and transcriptional activity. Mutations were densely clustered in the *NOTCH* signaling axis in a large number of regulators, highlighting its role in inappropriate transcriptional regulation. The changes in the *NRF2* pathway, which are less numerous, emphasized disturbance of the mechanisms of oxidative stress response. The *PI3K* pathway was commonly observed to be disrupted at several levels, such as receptor tyrosine kinases, *PI3K* catalytic subunits, and downstream transcription factors. One of the most comprehensive changes included *RTK-RAS* changes, including recurrent alterations in *FLT3*, *KRAS*, *KIT*, and *RAF* family members, as well as adaptor proteins, which indicates continuing proliferative signaling activation. TGF-b and the *TP53* pathway were disrupted, which was indicative of effects on immune regulation, the DNA damage response, and tumor suppressor signaling. Lastly, the *WNT* pathway was widely affected by mutations in regulators of canonical and non-canonical signaling, which is a sign of aberrant self-renewal and maintenance of stemness. Altogether, the pathway-level analysis shows that AML is defined by extensive and multi-pathway genomic alterations, which jointly reorganize cellular signaling, transcriptional programming, and proliferative controls. These changes are indicative of the polygenic and extremely interconnected nature of AML oncogenesis.

### 3.5. Functional Enrichment of Mutated Genes in AML

**Figure 5:**
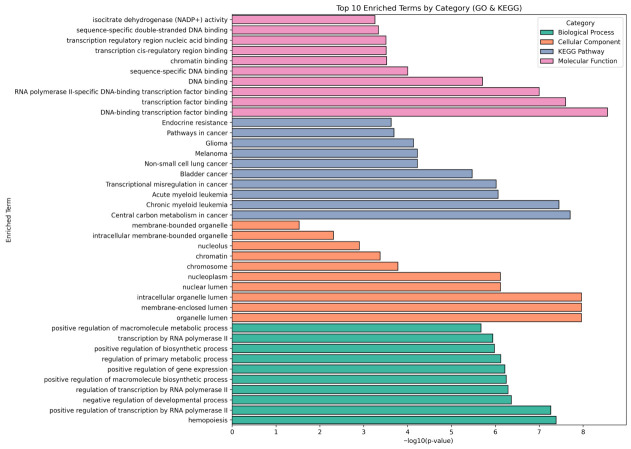
Functional enrichment analysis of mutated genes. Bar plot showing top 10 significantly enriched GO and KEGG terms for biological processes, molecular functions, and cellular components, as well as pathway enrichment.

Functional enrichment analysis combining Gene Ontology and KEGG pathway annotations demonstrated a point of convergence of somatic mutations on important biological processes and molecular functions (Figure 5). The enrichment of molecular functions was largely controlled by DNA binding– and chromatin-related activities, such as transcription factor binding and interaction with cis-regulatory regions, which is indicative of widespread interference with transcriptional control. Increased *IDH1*/*IDH2* mutations were correlated with enrichment of isocitrate dehydrogenase (NADP+) activity, which is indicative of changed metabolism control. KEGG pathway analysis demonstrated that cancer-related pathways, such as AML, transcriptional misregulation in cancer, and central carbon metabolism are enriched strongly. Further pathway enrichment of other malignancy pathways indicated overlapping malignant oncogenic signaling. The cellular component analysis displayed a predominant enrichment of compartments related to the nucleus and chromatin, such as the nuclear lumen, intracellular organelle lumen, chromosome, nucleoplasm, and membrane-bounded organelles, whereas a biological process enrichment further supported the role of pathways involved in the regulation of transcription through RNA polymerase II, biosynthetic and macromolecular metabolic processes, positive regulation of gene expression, and hematopoiesis. Collectively, these results suggest that mutations related to AML generally disrupt transcriptional control, chromatin structuring, metabolic processes, and hematopoietic differentiation programs.

### 3.6. Mutational Signature Profiling Reveals Underlying Mutagenic Processes

**Figure 6:**
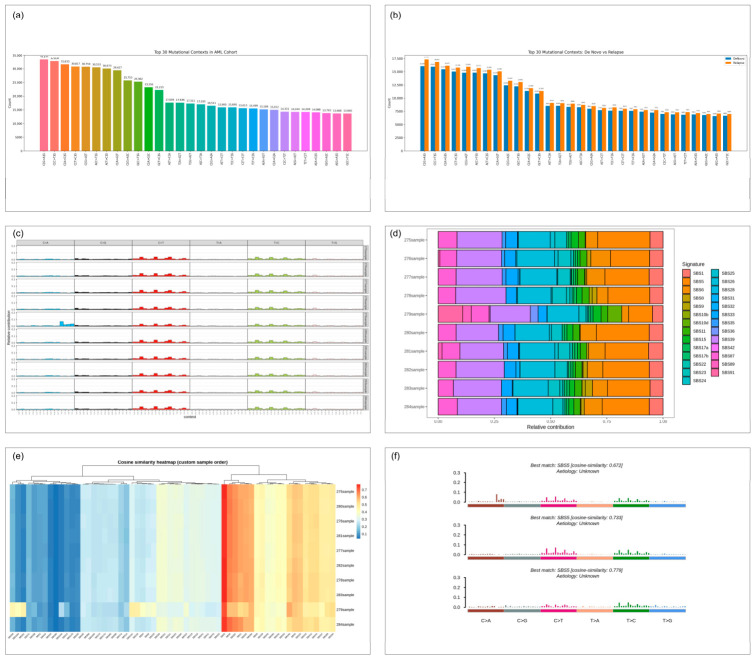
Mutational signature analysis in the AML cohort. (**a**) Bar plot displaying the 20 most common trinucleotide mutation contexts throughout the cohort. (**b**) Comparative bar plot of differences in the mutational context distribution of baseline (at diagnosis) and relapsed AML samples. (**c**) Panel depicting the 30 most common mutational multibase (MMB) contexts of samples. (**d**) Stacked bar plot of the relative contributions of the COSMIC SBS mutational signatures to each sample. (**e**) Cosine similarity heatmap that shows hierarchical clustering of samples with respect to reconstructed mutational signature profiles. (**f**) Signatures of representative COSMIC similarities between the values of cosine similarity and related etiologies.

Examination of trinucleotide mutational situations has shown prevailing enrichment of C > T replacement at CpG dinucleotides (Figure 6a), in accordance with endogenous cytosine deamination mechanisms. Further contributions of T > C and C > A substitutions implied the occurrence of a variety of mutagenic mechanisms. The comparison of baseline (at diagnosis) and relapse AML showed fairly similar substitution spectra, with a slight enrichment of specific C > T and T > C changes in the relapsed samples (Figure 6b). This relative enhancement implies that an increase in other mutational pressures throughout clonal evolution or following exposure to treatment, despite the fact that the overall mutational architecture held constant. Assessment of the six canonical SBS base replacement classes showed a stable dominance of C > T events in almost all of the samples (Figure 6c). Substitutions at lower frequencies such as C > A, T > A, T > G, and C > G, which played a relatively small role in mutagenesis in AML, reinforce the importance of the transition-type mechanisms in this context. When broken down into COSMIC reference signatures, individual contributions to each sample were found to occur by several mutational processes (Figure 6d). Signatures of SBS5 related to long-term endogenous DNA damage were present in virtually all individuals. SBS8, which has been shown to be involved with oxidative stress and thus involved in myeloid cancers, also played a significant role. Other signatures like SBS22, SBS24, SBS26, and SBS31 were present, but at lower levels, indicating that other processes were more dominant. The cosine similarity analysis was able to reconstruct mutational profiles (similarity > 0.8 in most cases) and provided clustering with a dominant contribution of SBS5/SBS40 or SBS8 (Figure 6e,f), indicating inter-patient heterogeneity in the mutational processes.

### 3.7. Drug–Gene Interaction Landscape and Druggability in AML

**Figure 7:**
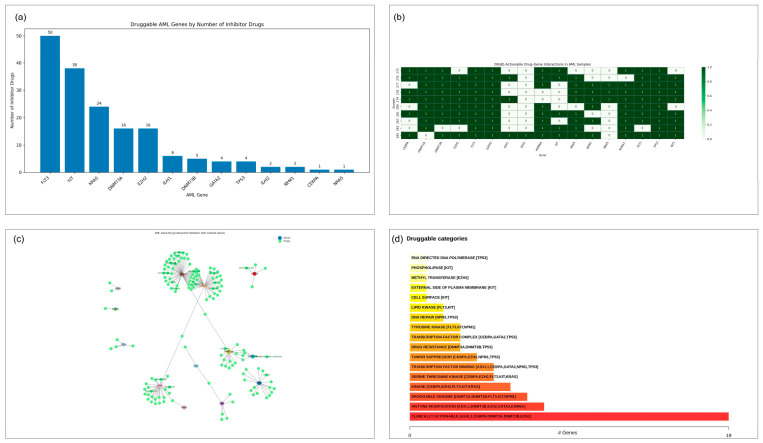
Drug–gene interaction landscape and druggability in AML. (**a**) The number of reported inhibitors against each recurrently mutated AML gene is plotted as a bar graph. (**b**) Heatmap of actionable drug–gene interactions across individual AML samples. (**c**) Network map of interactions between mutated AML genes and corresponding inhibitory compounds. (**d**) Therapeutic categories of druggable targets with key target classes indicated (including clinically actionable genes), such as kinases, methyltransferases, DNA repair proteins, and DNA repair proteins.

Recurrently mutated genes were analyzed for their druggability to determine the therapeutic potential of many classes of driver gene mutations. *FLT3* was the most druggable target, having 50 disease inhibitors, followed by *KIT* (38 disease inhibitors) and *KRAS* (24 disease inhibitors) (Figure 7a). *DNMT3A*, *EZH2*, *IDH1*, and *GATA2* were moderately druggable, and *CEBPA*, *NRAS*, and *NPM1* were only partially druggable. Mapped actionable drug–gene interactions in individual patient samples demonstrated heterogeneous therapeutic opportunities (Figure 7b). *FLT3*, *TP53,* and *GATA2* modifications accounted for most of the actionable hits, with several of the samples carrying several drug-responsive mutations. The distribution of actionable interaction counts was patient-specific and emphasized the unique therapeutic weaknesses in the cohort. Visualization of drug–gene interactions at the network level showed that there were clusters around large target families, such as kinases (*FLT3*, *KIT*, *KRAS*), epigenetic modifiers (*DNMT3A*, *EZH2*), and transcriptional controllers (*CEBPA*, *GATA2*, *TP53*) (Figure 7c). The central role of *FLT3* and *KIT* in drug-responsive hubs was highlighted by the presence of hub-like structures around them. Interactions between two or more epigenetic regulators indicated the possibility of convergent therapeutic interventions that can regulate chromatin structure and methylation pathways. Functional classification revealed the enrichment of pharmacologically tractable groupings such as tyrosine kinases, methyltransferases, DNA repair proteins, and clinically actionable targets (Figure 7d). These results indicate a rich and complex therapeutic environment, with the possibility of targeted intervention.

### 3.8. Experimental Validation of the Recurrent TET1 A256V Variant

**Figure 8:**
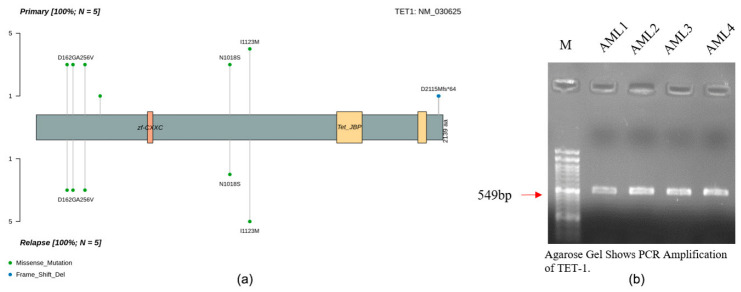
Confirmation of recurrent mutation of *TET1* in AML samples. (**a**) Lollipop plot of the presence of *TET1* mutations in baseline (at diagnosis) and relapsed samples of AML in functional domains (zf-CXXC, Tet JBP). The missense mutations appear in green, whereas the frame deletions are in blue. (**b**) Agarose gel electrophoresis gel showing PCR amplification of *TET1* in representative AML samples (red arrow, DNA ladder).

Protein domain mapping showed that *TET1* mutations were situated in major functional domains, such as the zf-CXXC DNA binding and Tet_JBP catalytic domains (Figure 8a). Missense mutations, such as D162G, A256V, N1018S, and I1123M, were also recurrent in the initial and relapsed samples, suggesting that these changes persisted during the course of the disease. It is important to note that A256V localized in the N-terminal segment of *TET1* and appeared in paired baseline (at diagnosis) and relapsed cases, which suggests its consistent detection across samples; however, its biological significance remains uncertain, given its high population frequency. The *TET1* A256V variant was experimentally validated by performing PCR amplification of the *TET1* exon 6 containing A256V, which generated a specific amplicon in all the samples analyzed (Figure 8b).

**Figure 9:**
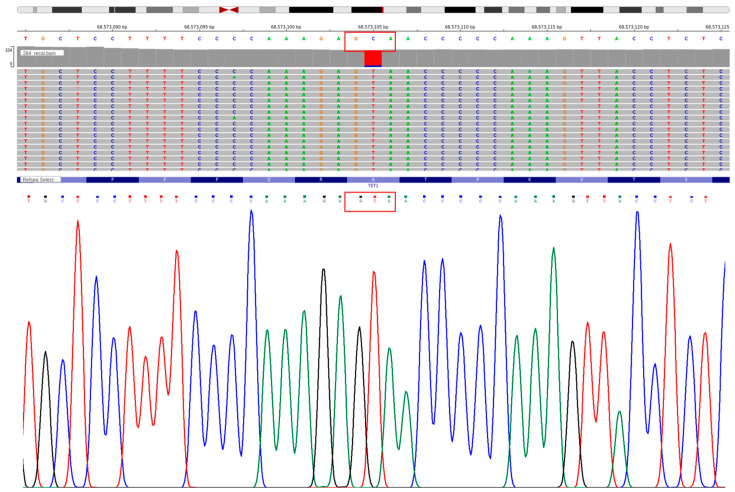
IGV and Sanger sequencing validation of the *TET1* A256V variant. The c.767C > T substitution at chr10:68,573,105 was confirmed by both methods, resulting in an A256V amino acid change.

Exome sequencing read inspection with IGV validated high-quality c.767C > T substitution and equal allele representation. The change was independently confirmed using Sanger sequencing, which revealed a definite change of C to T, which is equivalent to the A256V amino acid mutation (Figure 9). The *TET1* A256V variant (rs12221107) was recurrently observed across samples and validated experimentally. However, population database analysis (gnomAD) indicates that this variant has a relatively high allele frequency (~9% globally and ~18% in South Asian populations), suggesting that it represents a common germline polymorphism rather than a disease-specific somatic driver mutation.

### 3.9. TET1 Expression Analysis

**Figure 10:**
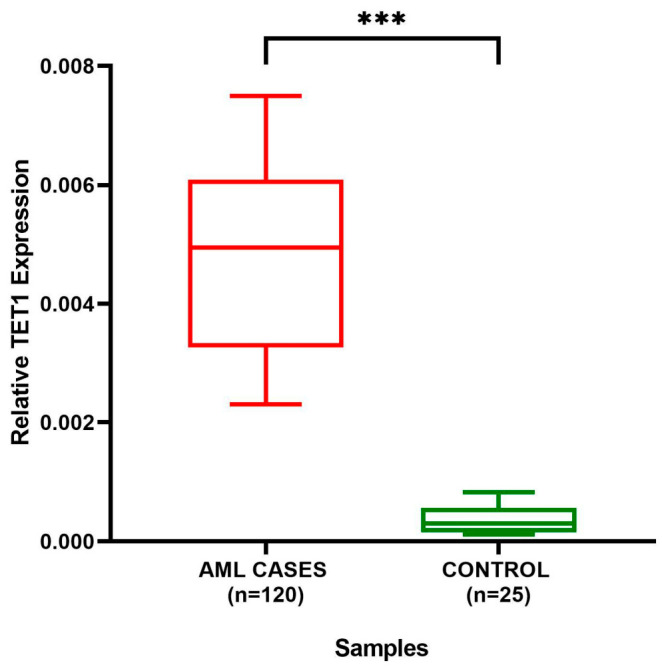
*TET1* expression levels in AML patients and healthy controls. ***: *p* < 0.001.

Quantitative real-time PCR analysis revealed a significant upregulation of *TET1* expression in AML patient samples compared to healthy controls. The median expression level of *TET1* was markedly higher in AML samples, with a wider interquartile range indicating variability among patients, whereas control samples exhibited consistently low expression levels (Figure 10). Statistical analysis demonstrated that this difference was highly significant (*p* < 0.001), indicating robust overexpression of *TET1* in AML. These findings suggest a potential role of *TET1* in AML pathogenesis and highlight its relevance as a potential biomarker for disease progression.

## 4. Discussion

AML is a heterogeneous hematological malignancy characterized by clonal proliferation of hematopoietic progenitors and accumulation of myeloblasts in the bone marrow, driven by genetic and epigenetic alterations [30]. Although induction chemotherapy effectively reduces leukemic burden in a large number of patients, there is still a high rate of relapse, and therefore there is a pressing need to develop better therapeutic approaches [31]. Sharing a similar molecular pattern, but with some distinct features in comparison with global cohorts of AML, we introduce an integrative genomic characterization of Indian AML patients with the use of whole-exome sequencing. Through mutual analysis of somatic single-nucleotide variants and mutational signatures, we were able to find recurrently mutated genes across epigenetic, transcriptional, and signaling pathways, and also specific patterns linked with disease relapse. The most common type of mutation was missense mutation, and common mutations were found in *TET1*, *FLT3*, *TP53*, *RUNX1*, *DNMT3A*, and *TET2*. These results align with mass AML sequencing studies, which indicate recurrent mutations in epigenetic regulators, signaling genes, and tumor suppressors and report four to five driver mutations per patient on average [32]. Notably, the *TET1* A256V variant identified in this study corresponds to rs12221107, which is reported at high frequency in population databases such as gnomAD, particularly in South Asian populations (~18%). In addition, in silico prediction tools (PolyPhen, REVEL) suggest a benign functional impact. Therefore, its recurrence in our cohort likely reflects underlying population genetics rather than a pathogenic role in AML. This highlights the importance of integrating population frequency data and germline filtering in the interpretation of genomic variants. In line with this, we found recurrent mutations in already known AML driver genes, such as *DNMT3A*, *TET2*, *FLT3*, *NPM1*, *RUNX1*, *IDH1*/*IDH2*, *ASXL1*, and *TP53*, which are central to leukemogenesis due to their functions in transcriptional regulation, DNA methylation, and chromatin remodeling [15,33,34]. The presence of *DNMT3A* and *TET2* mutations in most instances strengthens their role in the early stages of leukemogenesis and their association with clonal hematopoiesis of indeterminate potential (CHIP) [35,36,37,38]. It is noteworthy that *TET1* mutations occurred both in the baseline (at diagnosis) and relapsed samples. Though not as well-investigated as *TET2*, there is emerging evidence that *TET1* dysregulation changes the distribution of 5-hydroxymethylcytosine and affects the expression of genes, stem cell survival, and the hematopoietic differentiation process [39,40]. New findings also show that *TET1* mutation or overexpression could be involved in the pathogenesis of leukemia and poor clinical prognosis, which highlights its possible applicability in the biology of AML [41,42]. Our cohort of *TP53* mutations that were found to be enriched in relapsed samples is consistent with prior reports suggesting that *TP53* disruption causes genomic instability, resistance to therapy, and unfavorable prognosis [43]. The presence of *RUNX1*, *ASXL1*, and *EZH2* mutations also justifies the categorization of these three markers as adverse-risk markers in the ELN 2022 guidelines [44,45]. Moreover, frequent co-occurrence of *FLT3* and *NPM1* mutations was observed, indicating a relationship with disease progression and prognosis [46]. The higher incidence of *FLT3* mutations in relapsed samples in our dataset is similar to the findings of earlier studies, which indicate selective enrichment of *FLT3* mutant clones in response to chemotherapy [47,48]. Epigenetic modifications are of primary importance in AML biology. Mutations in *DNMT3A*, *TET1*/2, *IDH1*/2, *ASXL1,* and *EZH2* all influence DNA and histone methylation states in a way that causes deregulation of gene expression [49,50]. It has been shown that *DNMT3A* mutations disrupt de novo DNA methylation, thereby stimulating self-renewal and inhibiting differentiation of hematopoietic progenitors [51,52]. Likewise, mutations in *IDH1*/2 cause build-up of the oncometabolite 2-hydroxyglutarate, which suppresses TET-mediated hydroxymethylation and histone demethylases and create a hypermethylated phenotype [53]. These data highlight the importance of epigenetic disruption in the initiation and maintenance of AML and may help justify the assessment of hypomethylating agents or epigenetic modulators in particular patient groups.

Mutational signature analysis showed that the SBS5, SBS8, and SBS40 signatures were age-related and dominated by endogenous mutagenic events. Analysis of the mutational signatures in our cohort revealed an enrichment in C > T transitions at CpG dinucleotides, which is in line with spontaneous deamination of 5-methylcytosine being the most common endogenous mutational mechanism in AML [54]. This observation correlates with earlier large-scale studies, which have found the age-associated mutational processes SBS1 and SBS5 to be significant contributors to the AML mutational burden. In addition, relapsed samples had increased mutation loads and genome complexity compared to baseline (at diagnosis) cases, which suggests therapy-induced mutagenesis and clonal selection. According to the emerging literature, oxidative DNA damage and defective repair are caused by chemotherapy exposure, which further defines relapse-related mutational signatures [55]. All of these data are consistent with a sequential clonal evolution model of AML in which pre-leukemic or therapy-tolerant subclones obtain new genetic lesions that lead to relapses. From a translational viewpoint, our drug–gene interaction network revealed 19 gene targets that could be actionable, including *FLT3*, *KIT*, *KRAS*, *DNMT3A*, *EZH2,* and *TP53*, and *FLT3* had the most known number of inhibitors with potential therapeutic interest. *FLT3*-mutated AML can be treated with tyrosine kinase inhibitors like gilteritinib or quizartinib, which have shown clinical effectiveness in relapsed/refractory cases [56]. Mutated *IDH1* and *IDH2* can be inhibited using the small-molecule drugs ivosadenib and enasidenib, respectively, to restore normal differentiation programs [57]. Similarly, AML with *TP53* mutations, despite its conventional resistance to standard treatment options, can be targeted by the new agents like eprenetapopt (APR-246) or anti-CD47 therapy [58]. *DNMT3A* and *TET2* mutations can make patients vulnerable to hypomethylating agents (e.g., azacitidine, decitabine), especially when used in combination with a BCL2 inhibitor such as venetoclax, which has demonstrated effective synergy with epigenetically dysregulated AML [59]. Collectively, these molecular insights provide a foundation for precision oncology approaches in Indian AML patients, where mutation-guided therapy selection remains underexplored.

There are, however, some limitations. A major limitation of this study is the small cohort size (*n* = 10), which restricts the ability to draw statistically robust conclusions regarding mutation frequencies, co-occurrence patterns, and relapse-specific genomic signatures. Observations such as the apparent high frequency of alterations in genes including *TP53*, *TET1*, and *FLT3* are likely influenced by sampling effects and should not be interpreted as reflective of their true prevalence in AML. Furthermore, comparisons between baseline (at diagnosis) (*n* = 5) and relapsed (*n* = 5) samples are inherently underpowered, and observed differences, including mutation enrichment patterns such as that seen in *KRAS*, may not be reproducible in larger cohorts. Importantly, large-scale AML genomic studies have reported lower mutation frequencies for several of these genes, further supporting cautious interpretation of the current findings. Though our cohort was small, high-depth exome sequencing allowed the detection of SNVs with high strength, which gave us a holistic picture of SNVs in AML genomic modifications. Another limitation of this study is the lack of comprehensive integration of clinical parameters such as age, cytogenetic risk stratification, and prior treatment history, which may influence mutational patterns and clinical outcomes. Additionally, the absence of matched germline (non-tumor) controls represents a limitation, as complete discrimination between somatic and germline variants cannot be guaranteed despite stringent bioinformatic filtering. Further validation of new variants and transcriptomic integration (e.g., RNA-seq) would lend greater credence to mechanistic interpretations. Future research with regards to larger groups of patients and multi-omic integration with methylation, transcriptomic, and single-cell analyses will be required to validate our AML subclassifications and reveal new therapeutic vulnerabilities, specifically in the context of Indian populations. Altogether, our work identifies the mutational signature and clonal character of Indian AML patients and identifies common changes in epigenetic and signaling pathways, which mirror and expand on global AML findings. The dynamics of AML evolution are highlighted by enrichment of risky mutations and augmented genomic complexity, as well as by relapsed disease. These results serve as a genomic reference base and can facilitate the implementation of molecular profiling in normal clinical practice to enhance precision medicine practice in AML. These findings provide a foundational genomic reference and support the integration of molecular profiling into routine clinical workflows to advance precision medicine approaches in AML.

## 5. Conclusions

In this study, an integrative genomic analysis of AML was performed, giving clear insight into specific mutational and pathway-level changes in relation to disease progression and relapse. The frequent occurrence of *TET1*, *TP53*, *FLT3*, and *RUNX1* mutations indicates their role as key contributors in AML pathogenesis. Validation of the *TET1* A256V variant underscore its importance as a recurrently observed variant whose functional relevance remains uncertain. Functional enrichment and mutational signature studies underline the role of epigenetic deregulation and age-associated mutagenesis in the AML genome. Notably, actionable drug–gene interactions involving *FLT3*, *EZH2*, *DNMT3A*, and *KRAS* were identified, yielding potential avenues for personalized therapeutic interventions. While this study identifies potential genomic alterations and pathways involved in AML progression and relapse, the findings are exploratory and require validation in larger, independent cohorts. Overall, these results contribute to the knowledge of molecular pathways involved in AML and provide ground work to incorporate genomic profiling into precision medicine strategies to enhance patient outcomes.

## Data Availability

The data generated and analyzed during this study are available from the corresponding author upon reasonable request. Due to ethical considerations and restrictions outlined by the study’s approval and the informed consent obtained from participants, access to the data is limited. However, the datasets produced in this research have been deposited in the NCBI repository under BioProject ID PRJNA1223666.

## Abbreviations

The following abbreviations are used in this manuscript:

AML	Acute myeloid leukemia
BM	Bone marrow
NGS	Next-generation sequencing
GATK	Genome analysis toolkit
SNV	Single-nucleotide variants
GO	Gene Ontology
BP	Biological process
MF	Molecular function
CC	Cellular component
CHIP	Clonal hematopoiesis of indeterminate potential
